# A Contrast-enhanced Ultrasound Grading of Lymphatic Vessels: A Correlative Study and A Therapeutic Suggestion to Secondary Limb Lymphoedema

**DOI:** 10.2174/0115734056354068250415084500

**Published:** 2025-04-30

**Authors:** Ping Fu, Jia Zhu, Zijie Liu, Shentao Zhang, Shahi Kishor, Li Chen, Zhengren Liu, Lili Zhang

**Affiliations:** 1 Department of Ultrasound, The 1st Affiliated Hospital, Jiangxi Medical College, Nanchang University, No.17 Yong Wai Zheng Street, Nanchang, 330006, China; 2 Department of General Surgery, The 1st Affiliated Hospital, Jiangxi Medical College, Nanchang University, No.17 Yong Wai Zheng Street, Nanchang, 330006, China; 3 Department of Endocrinology and Metabolism, The 1st Affiliated Hospital, Jiangxi Medical College, Nanchang University,No.17 Yong Wai Zheng Street, Nanchang, 330006, China

**Keywords:** Postoperative tumor, Secondary limb lymphoedema, Function of lymphatic vessels, Contrast-enhanced ultrasound grading of lymphatic vessels, Indocyanine green, Lymphography

## Abstract

**Background:**

Various methods have been employed to evaluate secondary limb lymphedema, each with its own set of limitations.

**Objectives:**

To delve into a novel approach to lymphatic grading, specifically utilizing enhanced ultrasound for assessing lymphatic function, to compensate for the shortcomings of other methods to some extent.

**Materials and Methods:**

The clinical and ultrasound data of 51 patients with secondary limb lymphedema from June 2022 to September 2023 were retrospectively analyzed. The characteristic ultrasound manifestations of all visualized lymphatic vessels were studied. A contrast-enhanced ultrasound grading of lymphatic vessels (Ceus-Clv) was formulated and applied to grade the 51 patients. The study also correlated Ceus-Clv with Campisi clinical stage, postoperative duration, and duration of edema.

**Results:**

Out of 51 patients, there were 19 cases of Ceus-Clv I, 10 cases of Ceus-Clv II, 19 cases of Ceus-Clv III, and 3 cases of Ceus-Clv IV. The correlation coefficient (rs) between Ceus-Clv and Campisi clinical stages was 0.958 (P < 0.001). Similarly, the correlation coefficient between Ceus-Clv and postoperative duration was 0.824 (P < 0.001), and between Ceus-Clv and duration of edema was 0.763 (P < 0.001).

**Conclusion:**

Ceus-Clv grading is a safe, convenient, and effective method for assessing lymphatic vessel function in secondary limb edema. This method can accurately reflect the patient's lymphatic vessel function and the severity of edema, providing valuable guidance for the treatment of secondary limb edema.

## 1 INTRODUCTION

Secondary limb lymphedema is a prevalent postoperative complication of cancer, often seen in patients who have undergone surgery for malignant tumors like breast, prostate, cervical, ovarian, and endometrial cancers [1-5]. Globally, there are around 200 million individuals living with lymphedema, which can have lasting negative impacts on both physical and mental well-being [6, 7]. Early diagnosis and prevention of lymphedema are crucial for improving patients' quality of life [8]. Various methods, such as lymphoscin-tigraphy [9] to classify lymphatic patterns, magnetic resonance (MR) lymphography [10] for staging upper limb lymphedema, and indocyanine green (ICG) lymphography [11] for assessing lymphatic patterns, have been utilized. Tartaglione *et al*. [9] reported a classification of lymphatic patterns based on lymphoscintigraphy, but it is primarily used for initial assessments and preoperative guidance by identifying functional lymph nodes. However, its limitations include low resolution and inability to visualize peripheral vascular and interstitial tissues [12, 13]. Kim *et al*. proposed MR lymphography-based staging for upper limb edema. However, this method lacks consideration for tissue fibrosis and fat deposition, potentially leading to an underestimation of severity [10]. MR lymphography is a time-consuming and costly procedure that also requires high levels of patient cooperation, thus presenting limitations to its use in staging. ICG lymphography is the most commonly used modality in clinical practice [14]. Yamamoto *et al*. developed a staging of upper limb lymphoedema based on ICG lymphography [11]. Despite its widespread use, ICG lymphography has limitations in penetration depth, restricting its ability to provide a comprehensive view of the lymphatic system beyond 1.5 cm subcutaneously [15]. Contrast-enhanced ultrasound can be employed to address these limitations to some degree. Consequently, a novel approach for evaluating lymphatic vessels was developed through contrast-enhanced ultrasound to comprehensively assess the function of lymphatic vessels in the affected limb and assist in determining the appropriate clinical surgical strategy.

## 2 MATERIALS AND METHODS

### 2.1 Patients

From June 2022 to September 2023, enhanced ultrasound lymphography was conducted on a cohort of 51 female patients with secondary limb edema. All 51 patients were female, aged 34 to 71 years old. We retrospectively analyzed the clinical data and ultrasonographic manifestations of 51 patients.. 24 patients had upper limb edema following breast cancer surgery, 27 patients had lower limb edema following ovarian cancer or endometrial cancer surgery,11 of which had ovarian cancer and 16 had endometrial cancer. All 24 breast cancer patients underwent sentinel lymph node resection or axillary lymph node dissection,of which 7 underwent radiotherapy. All 27 patients with ovarian or endometrial cancer underwent pelvic lymph node dissection and none underwent radiotherapy. The postoperative period ranged from 5 to 382 months, and the duration of edema varied from 3 to 220 months. Patients with breast cancer had undergone lumpectomy and axillary lymph node dissection, with or without radiotherapy, while patients with cervical cancer or ovarian cancer had undergone uterine and bilateral adnexectomy along with inguinal lymph node dissection, also with or without radiotherapy.

The following patients were excluded: (1) those with severe cardiac and renal insufficiency; (2) those who have received treatment related to limb lymphedema; and (3) those who are unable to cooperate with the examination due to mental illness. All 51 patients were staged according to the Campisi clinical stage (Table **1**) [16], with 7 at stage 1, 9 at stage 2, 14 at stage 3, 18 at stage 4, and 3 at stage 5. All patients provided informed consent for participation in the study.

### 2.2 Instruments and Methods

The Siemens acuson sequoia ultrasonic diagnostic instrument was used in ultrasonography mode with probe 10L4, frequency 4~10MHz, and mechanical index 0.06~0.08. A higher frequency probe was not chosen because a higher frequency would destroy the microbubbles of the contrast agent. The contrast agent used was SonoVue lyophilized powder (Bracco company), SF6 microbubbles 59 mg in 5 ml of saline, which was shaken and prepared for use. The lymphangiography procedure was conducted by an experienced senior physician before surgery. Selection of the injection site for the contrast agent with reference to the indocyanine green injection point [11] and according to the alignment of the collecting lymphatic vessels,subcutaneous injection of multiple points in the area of their alignment, the contrast agent can be fully absorbed by all the collecting lymphatic vessels. In order to fully visualise all lymphatic vessels, patients with upper limb edema received an intradermal contrast agent injection at specific points in the affected limb, including space between each finger web, on the ulnar and radial side of both the ventral and distal surface of the wrist joint also on the medial and lateral sides of both the ventral and distal surface of the elbow joints. In the affected lower limb, injection points were chosen at each space between the toes, the anterior and posterior sides of both the inner and outer ankle, as well as the medial and lateral sides of the knee joints and popliteal fossa. Each injection point received 0.5 ml of suspension *via* a 25G injection needle, followed by a gentle massage for 5-15 seconds in order to keep the lymphatic vessels visualized so that we can see them adequately. If necessary, we will increase the dose of contrast agent to extend the observation time. The lymphatic vessels were then observed using ultrasonography by tracing along the long axis of the limb. The visualization of subcutaneous capillary lymphatic vessels was influenced by the contrast agent absorption at the injection site, requiring observation within a scope of more than 3cm from the injection point for accurate results.

**Fig. (1):**
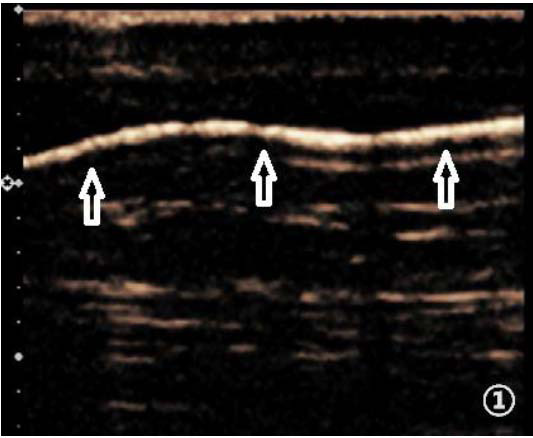
Linear collecting lymphatic vessels (indicated by arrows).

**Fig. (2):**
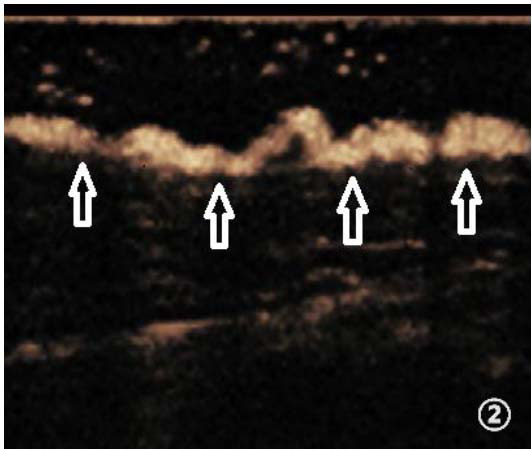
Tortuous and dilated lymphatic vessels (indicated by arrows).

**Fig. (3):**
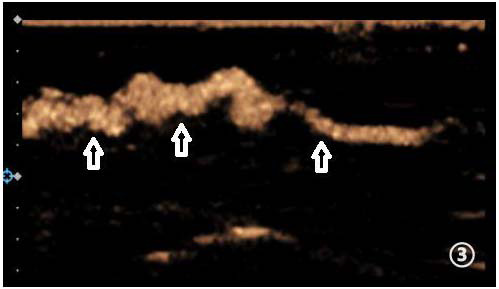
Lymphatic vessels with uneven internal diameters (indicated by arrows).

**Fig. (4):**
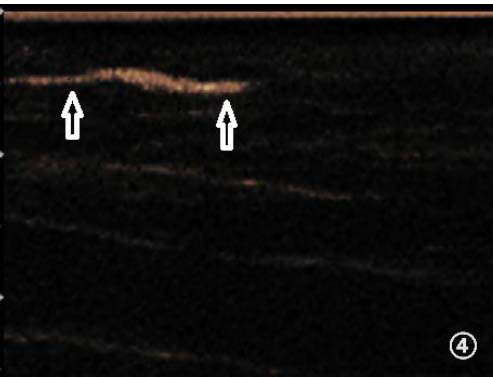
Short rod shapes (indicated by arrows).

**Fig. (5):**
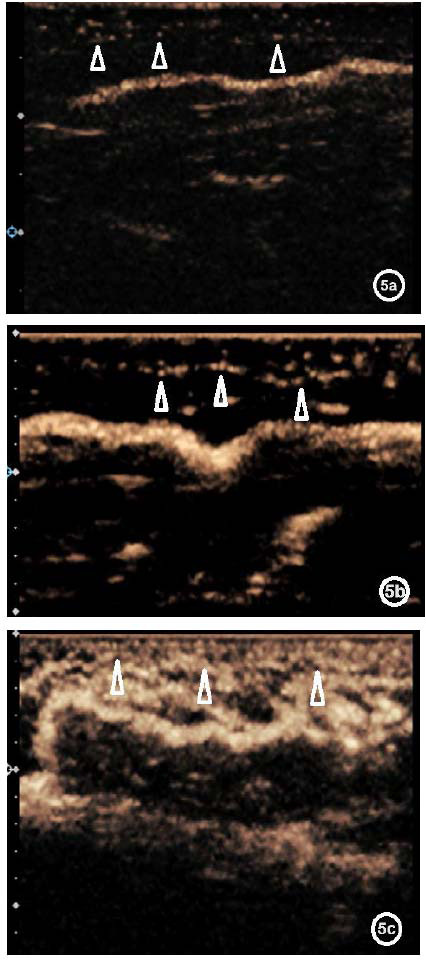
Visible subcutaneous capillary lymphatics. **5a**: Mild dilatation of subcutaneous capillary lymphatics (indicated by triangles), with some enhancement showing a stellate distribution. **5b**: Increased dilatation of subcutaneous capillary lymphatics (indicated by triangles), showing both stellate and a linear distribution. **5c**: Marked dilatation of subcutaneous capillary lymphatic vessels (indicated by triangles) , showing a diffuse distribution.

**Fig. (6):**
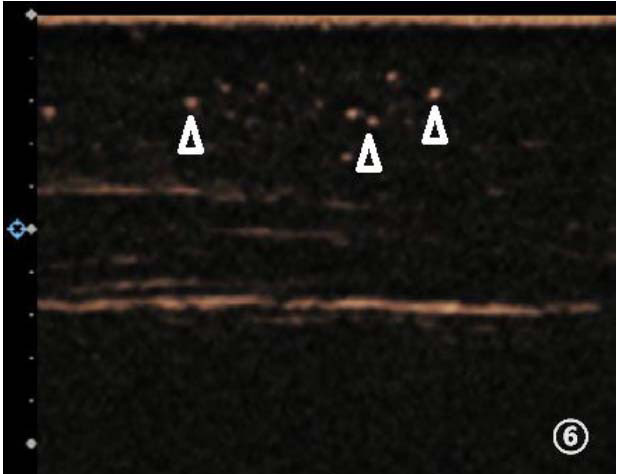
Collecting lymphatic vessels were solely short rods or not visible, with only stellate contrast bubbles observed subcutaneously (indicated by triangles).

We used contrast-enhanced ultrasound for all lymphatic evaluation because two-dimensional ultrasound has very limited utility for lymphatic evaluation. Following enhanced ultrasonography, a detailed analysis of the visualized lymphatic vessels in the patient was conducted and categorized into four grades. Ceus-Clv I indicates linear collecting lymphatic vessels, implying excellent lymphatic vessel function. Ceus-Clv II shows any of the lymphatic vessels displaying only one of the following signs, like refluxed, tortuous and dilated lymphatic vessels, lymphatic vessels with uneven internal diameters or short rod shapes, visible subcutaneous capillary lymphatics (Figs. **1**-**5**). It indicates good lymphatic vessel function. Ceus-Clv III shows any of the lymphatic vessels displaying two of the above signs, indicating poor lymphatic vessel function. Ceus-Clv IV features collecting lymphatic vessels that are solely short rods or not visible, with only stellate contrast bubbles observed subcutaneously (Fig. **6**), indicating poor lymphatic vessel function.

### 2.3 Statistical Methods

The data were analyzed using IBM SPSS 25.0. Spearman's correlation was employed to examine the relationship between Campisi's clinical stage, postoperative duration, duration of edema, and Ceus-Clv. A p-value below 0.05 was deemed statistically significant. A positive rs value denoted a positive correlation, a negative value denoted a negative correlation, and a value of zero indicated no correlation. Correlation coefficients with an absolute value between 0.8 and 1.0 indicate a very strong correlation between variables. An absolute value between 0.6 and 0.8 indicates a strong correlation, between 0.4 and 0.6 indicates a moderate correlation, between 0.2 and 0.4 indicates a weak correlation, and between 0.0 and 0.2 indicates a very weak or no correlation between variables.

## 3 RESULTS

Out of the 51 patients, 19 were graded as Ceus-Clv I, 10 as Ceus-Clv II, 19 as Ceus-Clv III, and 3 as Ceus-Clv IV. The study revealed that within Ceus-Clv I, there were 7 cases of Campisi clinical stage 1, 9 cases of stage 2, and 3 cases of stage 3. For patients in Ceus-Clv II, there were 9 cases of Campisi clinical stage 3 and 1 case of stage 4. Among those in Ceus-Clv III, 2 cases of Campisi clinical stage 3, 17 cases of stage 4 were observed, while all 3 patients in Ceus-Clv IV had Campisi clinical stage 5 (Table **2**).

Among patients with lymphatic contrast-enhanced ultrasound grading Ceus-Clv II and Ceus-Clv III, 24 cases (24/29) had lymphatic reflux, and 18 cases (18/29) had subcutaneous lymphatic capillary imaging. The tortuous expansion of collecting lymphatic vessels was only seen in Ceus-Clv III (5/19), the collecting lymphatic vessels were short rod-shaped and were only seen in Ceus-Clv III and Ceus-Clv IV Table **3**.

**Fig. (7):**
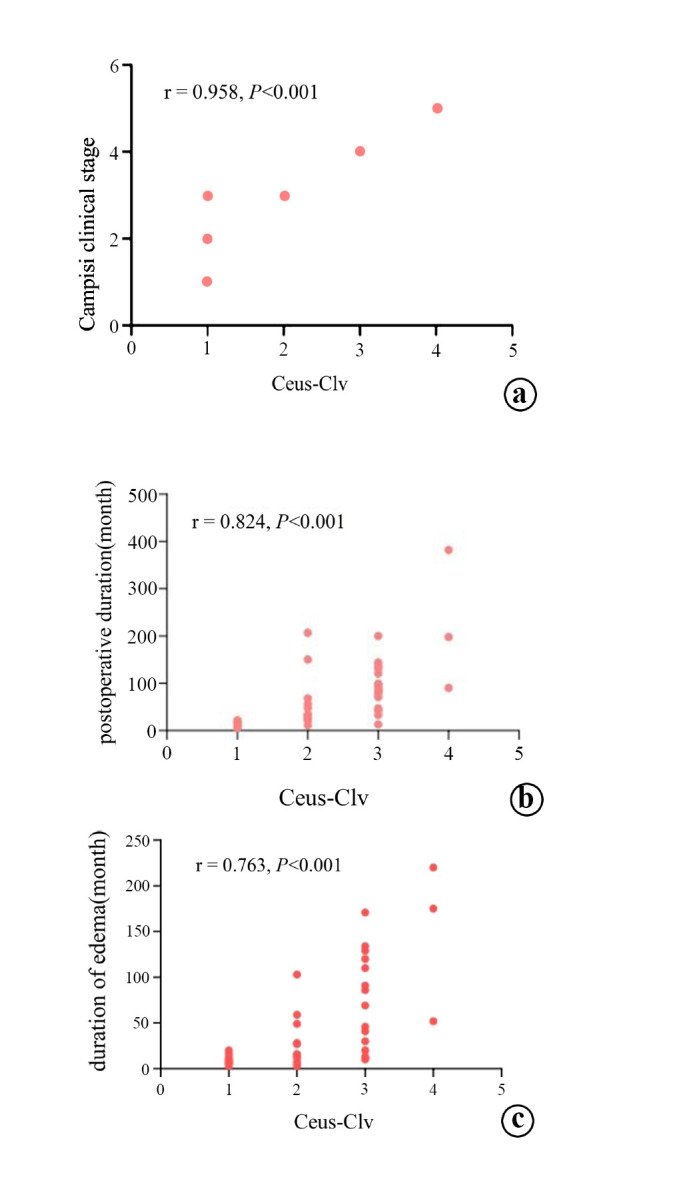
The correlation coefficient (rs) values between Ceus-Clv and Campisi clinical stage, postoperative duration, and duration of edema were 0.958 (**a**), 0.824 (**b**), and 0.763 (**c**), respectively.

The correlation coefficient (rs) values between Ceus-Clv and Campisi clinical stage, postoperative duration, and duration of edema were 0.958 (P < 0.001), 0.824 (P < 0.001), and 0.763 (P < 0.001), respectively (Fig. **7**).

Out of the 51 patients with limb edema, 29 patients were graded as Ceus-Clv II and III., and we recommended Lymphatic venous anastomosis (LVA), which is a minimally invasive surgical procedure for treating lymphedema [17, 18]. 19 patients underwent LVA, resulting in the improvement of their edema post-procedure.

## 4 DISCUSSION

Lymphatic vessels play a crucial role in transporting lymphatic fluids. In cancer patients, post-surgery obstruction of lymphatic fluid flow can lead to the accumulation of protein-rich fluids in the tissues, causing edema [19]. Lymphedema is characterized by edema, inflammation, and cellulitis, resulting in symptoms such as limb swelling, pain, numbness, limited mobility, and cosmetic issues with the skin. This significantly impacts the quality of life of patients.

Conservative treatment is an option for patients with mild symptoms, while LVA or can be used for severe edema patients. The accurate assessment of lymphatic vessel function is essential for successful LVA. Various methods are used for lymphatic vessel assessment, each with its limitations. Therefore, there is a need to explore new approaches.

With advancements in ultrasound technology, researchers have moved beyond identifying lymphatic vessels to assessing their function. In 2016, Hayashi, Czedik-Eysenberg, Hara, *etc*. [20-22]. reported that high-frequency ultrasound could be used to identify lymphatic vessels and determine their location. Mihara *et al*. [23] reported that high-frequency ultrasound can be used to determine the degree of degeneration of lymphatic vessels. Jang *et al*. [24]and Xiahou *et al*. [25] have shown that lymphatic contrast-enhanced ultrasound is more intuitive and better at developing lymphatic vessels and can observe the peristalsis direction of lymphatic vessels, the degree of expansion, and the distribution of lymphatic capillaries, thereby judging the function of lymphatic vessels. Therefore, we established a system based on contrast-enhanced ultrasound lymphatic grading, a comprehensive analysis, and grading of all developed lymphatic vessels in different patients.

We have found that as the disease progresses, the lymphatic function of patients with limb lymphedema gradually worsens. In patients with mild edema, only the collecting lymphatic vessels are developed and appear linear. At this time, the lymphatic function is excellent; as the disease progresses, lymphatic vessels may exhibit lymphatic valve insufficiency and lymphatic reflux. In longer cases, the collecting lymphatic vessels may expand tortuously, and the subcutaneous lymphatic capillaries will be visualized. In severe cases, the lymphatic vessel wall will be partially narrowed or occluded due to fibrosis, the inner diameter of the lymphatic vessels will be irregular or short rod shaped after enhanced ultrasonography. Collecting lymphatic vessels won’t be visualized but will appear as a subcutaneous spotty pattern. We performed a comprehensive analysis of all developing lymphatic vessels in different patients and graded them into Ceus-Clv I~ IV.

The results of this study show that Ceus-Clv has a very strong correlation with Campisi clinical stage and postoperative duration, and a strong correlation with the duration of edema. Most of the patients with Ceus-Clv I were in Campisi stage 1 or 2, and only 3 cases were in stage 3. The postoperative duration of these 3 patients was short, all less than 12 months; while most of the patients with Ceus-Clv II were in Campisi stage 3, only one case was in Campisi stage 4, and the postoperative time was 13 months. Ceus-Clv III was mostly in Campisi stage 4, and 2 cases were in Campisi grade 3. One of these two patients had lymphatic reflux and subcutaneous capillary lymphatic imaging; In addition to the reflux of lymph fluid, short rod-shaped lymphatic vessels appeared in another case due to localized erysipelas. The 3 patients with Ceus-Clv IV were all in Campisi stage 5. In short, the higher the clinical edema stage, the longer the postoperative duration, and the higher the lymphatic contrast-enhanced ultrasound grade. However, compared with the postoperative duration, the correlation with the duration of edema is relatively low. In our analysis, it may be because the symptoms of early edema are not obvious, and some patients cannot be identified, but the actual duration of edema should be longer.

Ceus-Clv I is more common in patients with mild edema, Ceus-Clv II and III are more common in patients with more severe edema, and Ceus-Clv IV is more common in patients with severe edema. For patients with lymphatic contrast-enhanced ultrasound grading of Ceus-Clv I, we recommend conservative treatment; for Ceus-Clv II and Ceus-Clv III, we recommend LVA surgery; for Ceus-Clv IV, the lymphatic function is extremely poor. Surgery is currently not possible. For patients recommended for surgery, we can also screen out lymphatic vessels with relatively good functions for pre-operative positioning to improve surgical efficacy. 19 patients underwent lymphovenous anastomosis, and their edema symptoms improved after surgery.

## CONCLUSION

This result shows that CEUS allows better visualization of limb lymphatics, and Ceus-Clv grading can well reflect the lymphatic function of patients with secondary limb lymphedema and provide visual imaging information for oedema patients. As such, it provides a reliable basis for the selection of treatment options for such patients. Ceus-Clv grading is safe, radiation-free, effective, requires low patient cooperation, and can also develop deep lymphatic vessels very well, which can make up for the shortcomings of other methods to a certain extent.

This study also has some shortcomings. Because the sample size of our study is small, the results may have certain biases. In the future, we will collect more edema patients and increase the sample size.

## Figures and Tables

**Table 1:** Staging of peripheral lymphedema based on clinical patterns.

Stage 1:
1A No edema, with presence of lymphatic dysfunction
1B Mild edema, reversible with elevation of the limb and night rest
Stage 2: Persistent edema that regresses only partially with elevation of the limb and night rest
Stage 3: Persistent edema that continually becomes more severe (recurrent acute erysipeloid lymphangitis)
Stage 4: Fibrotic lymphedema (with initial lymphostatic warts) and column-shaped limb
Stage 5 Elephantiasis with severe limb deformation, scleroindurative pachydermitis, and widespread lymphostatic warts

**Table 2:** Ceus-Glv and Campisi clinical stage.

Ceus-Glv/ Campisi clinical stage	Stage 1	Stage 2	Stage 3	Stage 4	Stage 5	Total
I	7	9	3	0	0	19
II	0	0	9	1	0	10
III	0	0	2	17	0	19
IV	0	0	0	0	3	3
Total	7	9	14	18	3	51

**Table 3:** The lymphangiographic manifestations of Ceus-Clv I~IV.

	Ceus-Clv I	Ceus-Clv II	Ceus-Clv III	Ceus-Clv IV	Total
Linear collectinglymphatic vessels	19	-	-	-	19
Refluxed	-	8	16		24
Tortuous and dilated lymphatic vessels	-	-	5	-	5
Visible subcutaneous capillary lymphatics	-	2	16	-	18
Lymphatic vessels with uneven internal diameters or short rod shapes	-	-	2	-	2
Collecting lymphatic vessels are solely short rods or not visible, with only stellate contrast bubbles observed subcutaneously	-	-	-	3	3

## Data Availability

All the data and supporting information are provided within the article.

## LIST OF ABBREVIATIONS

MR: Magnetic resonance
ICG: Indocyanine green
LVA: Lymphatic venous anastomosis

## STANDARDS OF REPORTING

STROBE guidelines were followed.

## References

[1] Zou L., Liu F., Shen P., Hu Y., Liu X., Xu Y., Pen Q., Wang B., Zhu Y., Tian Y. (2018). The incidence and risk factors of related lymphedema for breast cancer survivors post-operation: A 2-year follow-up prospective cohort study.. Breast Cancer.

[2] Warren A.G., Brorson H., Borud L.J., Slavin S.A. (2007). Lymphedema: A comprehensive review.. Ann. Plast. Surg..

[3] Saha M., Edmonds E., Martin J., Bunker C.B. (2009). Penile lymphoedema in association with asymptomatic Crohn’s disease.. Clin. Exp. Dermatol..

[4] McLaughlin S.A., Wright M.J., Morris K.T., Sampson M.R., Brockway J.P., Hurley K.E., Riedel E.R., Van Zee K.J. (2008). Prevalence of lymphedema in women with breast cancer 5 years after sentinel lymph node biopsy or axillary dissection: Patient perceptions and precautionary behaviors.. J. Clin. Oncol..

[5] Abu-Rustum N.R., Alektiar K., Iasonos A., Lev G., Sonoda Y., Aghajanian C., Chi D.S., Barakat R.R. (2006). The incidence of symptomatic lower-extremity lymphedema following treatment of uterine corpus malignancies: A 12-year experience at Memorial Sloan-Kettering Cancer Center.. Gynecol. Oncol..

[6] Cheng M.H., Pappalardo M., Lin C., Kuo C.F., Lin C.Y., Chung K.C. (2018). Validity of the novel taiwan lymphoscintigraphy staging and correlation of cheng lymphedema grading for unilateral extremity Lymphedema.. Ann. Surg..

[7] Cemal Y., Pusic A., Mehrara B.J. (2011). Preventative measures for lymphedema: Separating fact from fiction.. J. Am. Coll. Surg..

[8] Gärtner R., Mejdahl M.K., Andersen K.G., Ewertz M., Kroman N. (2014). Development in self-reported arm-lymphedema in Danish women treated for early-stage breast cancer in 2005 and 2006 – A nationwide follow-up study.. Breast.

[9] Tartaglione G., Visconti G., Bartoletti R., Gentileschi S., Salgarello M., Rubello D., Colletti P.M. (2018). Stress lymphoscintigraphy for early detection and management of secondary limb lymphedema.. Clin. Nucl. Med..

[10] Kim G., Smith M.P., Donohoe K.J., Johnson A.R., Singhal D., Tsai L.L. (2020). MRI staging of upper extremity secondary lymphedema: Correlation with clinical measurements.. Eur. Radiol..

[11] Yamamoto T., Yamamoto N., Doi K., Oshima A., Yoshimatsu H., Todokoro T., Ogata F., Mihara M., Narushima M., Iida T., Koshima I. (2011). Indocyanine green-enhanced lymphography for upper extremity lymphedema: A novel severity staging system using dermal backflow patterns.. Plast. Reconstr. Surg..

[12] Nagy B.I., Mohos B., Tzou C.H.J. (2023). Imaging modalities for evaluating lymphedema.. Medicina.

[13] Liu M., Liu S., Zhao Q., Cui Y., Chen J., Wang S. (2022). Using the indocyanine green (ICG) lymphography to screen breast cancer patients at high risk for lymphedema.. Diagnostics.

[14] Imai H., Yoshida S., Mese T., Roh S., Fujita A., Sasaki A., Nagamatsu S., Koshima I. (2022). Correlation between lymphatic surgery outcome and lymphatic image-staging or clinical severity in patients with lymphedema.. J. Clin. Med..

[15] Zeltzer A.A., Anzarut A., Hamdi M. (2018). A review of lymphedema for the hand and upper-extremity surgeon.. J. Hand Surg. Am..

[16] Campisi C., Boccardo F. (2004). Microsurgical techniques for lymphedema treatment: Derivative lymphatic-venous microsurgery.. World J. Surg..

[17] Hara H., Mihara M. (2021). Lymphaticovenous anastomosis for advanced‐stage lower limb lymphedema.. Microsurgery.

[18] Mihara M., Hara H., Kawasaki Y., Mitsuhashi T., Orikasa H., Ando H., Naito M. (2024). Lymphatic venous anastomosis and complex decongestive therapy for lymphoedema: Randomized clinical trial.. Br. J. Surg..

[19] Forte A.J., Khan N., Huayllani M.T., Boczar D., Saleem H.Y., Lu X., Manrique O.J., Ciudad P., McLaughlin S.A. (2020). Lymphaticovenous anastomosis for lower extremity lymphedema: A systematic review.. Indian J. Plast. Surg..

[20] Hayashi A., Yamamoto T., Yoshimatsu H., Hayashi N., Furuya M., Harima M., Narushima M., Koshima I. (2016). Ultrasound visualization of the lymphatic vessels in the lower leg.. Microsurgery.

[21] Czedik-Eysenberg M., Steinbacher J., Obermayer B., Yoshimatsu H., Hara H., Mihara M., Tzou C.H.J., Meng S. (2020). Exclusive use of ultrasound for locating optimal LVA sites—A descriptive data analysis.. J. Surg. Oncol..

[22] Hara H., Mihara M. (2023). Ultrasound‐guided lymphaticovenous anastomosis without indocyanine green lymphography mapping: A preliminary report.. Microsurgery.

[23] Mihara M., Hara H., Kawakami Y. (2018). Ultrasonography for classifying lymphatic sclerosis types and deciding optimal sites for lymphatic-venous anastomosis in patients with lymphoedema.. J. Plast. Reconstr. Aesthet. Surg..

[24] Jang S., Lee C.U., Hesley G.K., Knudsen J.M., Brinkman N.J., Tran N.V. (2022). Lymphatic mapping using us microbubbles before lymphaticovenous anastomosis surgery for lymphedema.. Radiology.

[25] Xiahou Y., Yuan X., Zhu J., Hu W., Zhang L. (2023). The significance of contrast-enhanced ultrasound in the application of lymphaticovenous anastomosis.. Curr. Med. Imaging.

